# NKP-1339, a first-in-class anticancer drug showing mild side effects and activity in patients suffering from advanced refractory cancer

**DOI:** 10.1186/2050-6511-13-S1-A82

**Published:** 2012-09-17

**Authors:** Robert Trondl, Petra Heffeter, Michael A Jakupec, Walter Berger, Bernhard K Keppler

**Affiliations:** 1 Institute of Inorganic Chemistry, University of Vienna, 1090 Vienna, Austria; 2 Institute of Cancer Research, Medical University of Vienna, 1090 Vienna, Austria; 3 Research Platform Translational Cancer Therapy Research, University of Vienna, 1090 Vienna, Austria

## Background

NKP-1339 is one of the most promising investigational non-platinum metal drugs in clinical development against solid malignancies. Recently, NKP-1339 was evaluated in a clinical phase I trial regarding its safety, tolerability, maximum tolerated dose, pharmacokinetics, and pharmacodynamics. The high tumor targeting potential of NKP-1339 is probably based on delivery to tumor sites by serum proteins such as albumin and transferrin as well as on the activation of the compound in the reductive tumor milieu. The reduction of ruthenium(III) to ruthenium(II) is favoured under hypoxic condition (frequently occurring in solid tumors) and is followed by severe disruption of the cellular redox balance and induction of apoptosis via the mitochondrial pathway.

## Methods

In the recently completed clinical phase I trial 34 patients with advanced solid tumors were enrolled for dose escalation [1]. NKP-1339 was infused on day 1, 8, and 15 of 28-day cycles. To gain further insight in the mechanism of action, protein expression studies in cancer cells exposed *in vitro* were performed.

## Results

The maximum tolerated dose of NKP-1339 was determined at 625 mg/m^2^, and the most common drug-related side effects were nausea, vomiting, and fatigue. A long-lasting partial response was observed in 1 patient with a gastro-intestinal neuroendocrine tumor (NET), and 7 patients having experienced stable disease, including NET (2), non-small-cell lung cancer (2), colorectal cancer (1), sarcoma (1), and cancer of unknown primary (1). Moreover, NKP-1339 was found to down-regulate in cancer cell lines the ER chaperon GRP78, a key regulator of the unfolded protein response, which is associated with intrinsic and chemotherapy-induced resistance.

## Conclusions

The very limited side effects of NKP-1339 and the activity observed in a variety of tumors so far are very promising. Further clinical phase I drug combination studies and single agent phase II studies are planned.
